# FAIR research data management as community approach in bioengineering

**DOI:** 10.1002/elsc.202200005

**Published:** 2022-04-28

**Authors:** Martina Rehnert, Ralf Takors

**Affiliations:** ^1^ Institute of Biochemical Engineering University of Stuttgart Stuttgart Germany

**Keywords:** bioengineering, community approach, data sustainability, data management, FAIR

## Abstract

Research data management (RDM) requires standards, policies, and guidelines. Findable, accessible, interoperable, and reusable (FAIR) data management is critical for sustainable research. Therefore, collaborative approaches for managing FAIR‐structured data are becoming increasingly important for long‐term, sustainable RDM. However, they are rather hesitantly applied in bioengineering. One of the reasons may be found in the interdisciplinary character of the research field. In addition, bioengineering as application of principles of biology and tools of process engineering, often have to meet different criteria. In consequence, RDM is complicated by the fact that researchers from different scientific institutions must meet the criteria of their home institution, which can lead to additional conflicts. Therefore, centrally provided general repositories implementing a collaborative approach that enables data storage from the outset In a biotechnology research network with over 20 tandem projects, it was demonstrated how FAIR‐RDM can be implemented through a collaborative approach and the use of a data structure. In addition, the importance of a structure within a repository was demonstrated to keep biotechnology research data available throughout the entire data lifecycle. Furthermore, the biotechnology research network highlighted the importance of a structure within a repository to keep research data available throughout the entire data lifecycle.

**Abbreviations**
FAIR: findable, accessible, interoperable, re‐ssable
RDM: research data management

1

Research data management (RDM) is a complex and dynamic process that guides researchers through the various stages of the data lifecycle and enables scientists and other stakeholders to effectively and responsibly manage community‐generated research data [1]. The expected outcome should be findable, accessible, interoperable, and reusable (FAIR) research data, as previously proposed by Wilkinson et al. [2]. FAIR research data management consistently follows data through all seven life cycles: plan, collect, analyze, publish, preserve, share, and reuse [3] and therefore needs modern tools and the recognition by the scientific community. Therefore, the FAIR principles [2], adapted by research organizations such as RDA [4], recently also demanded and recognized by publishers as Nature Publisher group [5] and funders such as US National Institutes of Health NIH [6], or consortia like German NFDI [7] for the entire research data life cycle [3].

However, modern tools and techniques for managing research data are still hesitantly used by the research community [8]. Public databases and repositories as well as registries are available for FAIR handling of data, which are also increasingly demanded by publication journals for handling data [5]. While databases are mostly used on a topic‐specific basis, repositories serve for sustainable and FAIR data management of research data by providing data with digital identifiers [9]. Repository selection is facilitated by registries such as re3ata, FAIRsharing, DataCite [10]. However, repositories can also be specialized in certain areas, as known from databases such as NCBI's gene sequence database GenBank [11]. In contrast, general repositories such as Dataverse, Dryad, Figshare, OSF, and Zenodo are open to all disciplines [12] and are suitable for discipline independent research data throughout its lifecycle.

Investigations suggests that there is often irritation for researchers what research data sharing means and how terms such as “data repositories, FAIR and RDM” should be understood [13]. Many researchers equated data storing in repositories with data sharing [14]. Researchers expressed concerns such as lack of support and infrastructures or worries about misinterpretation and misuse of shared data [13, 14]. Managing research data is time‐consuming, costly, and tedious, requiring additional resources that are not available to all researchers [13, 14]. Data managers who take over and are responsible for support, data maintenance, and infrastructure deployment are not provided in every research department [15].

**PRACTICAL APPLICATION**

Due to the huge and growing amount of data, the importance of findable, accessible, interoperable, and reusable (FAIR) research data management (RDM) is becoming increasingly important, but is still rather hesitantly considered in bioengineering. One of the reasons may be the specific character of bioengineering, where two disciplines – life sciences and engineering – have to meet different criteria and have to fulfil different expectations of the communities. Here, we present the example of a bioengineering research network with tandem projects each comprising researchers of the two disciplines. It is demonstrated, how important a structure within a data repository is, to keep research data available throughout the entire data life cycle.

Today's data driven science community, publishers, and funders rely on sustainable data [16]. Research data management has to consider that stakeholders such as developers, data curators, collaborative researchers, community managers, and research network coordinators are mostly decentralized at different institutions with different funding and infrastructure [16, 17, 18]. Researchers need to implement the criteria of their own local home institutions rather than global requirements for data storage in databases or repositories, which requires duplicate storage [17]. Even when repositories meet all *FAIR* criteria, conflicts can arise because data management with duplicate data storage can be confusing and requires more resources [14, 15]. Each data user, data creator, or data curator, has different needs and perspective. Researchers in academia compete for funding and publications; on the one hand, they need networks, but on the other hand, they are afraid of losing control on their data [19]. Funders, data policy makers, and publishers benefit because they get better perspective and reporting and coverage [5, 16, 19].

RDM as a collaborative approach and team effort can address issues such as the need for a sustainable research data structure needed by all stakeholders and required by publishers, funders, or policy makers [19, 20, 21]. Any strategy that makes data appropriately available for grant applications, publication, or sustainable reuse demonstrates the benefits of data management [13, 19]. Decision makers pursue different strategies in this regard, while a top‐down strategy is more appropriate for designing the data structure, a bottom‐up strategy is more effective for data storage [8, 15, 21]. Often, researchers perform data management too late in the data life cycle, which means additional effort and data may not be of adequate quality [13, 14]. Therefore, each community needs coordinators to consider the various requirements early in the data life cycle. Structural issues such as data security, duration of data storage, dynamization, selection of databases and repositories, sharing data or making data available for publication, and compliance with legal and timing requirements are just some of the issues that are often evaluated differently from different perspectives [20, 21]. Sustainable RDM is only feasible and FAIR if all stakeholders have a common understanding and approach [2, 20].

In life sciences, as a part of biotechnology, standards and more than 400 databases and repositories are available for research community [5, 11]. Electronic laboratory journals improve data processing and documentation from the beginning of the experiment in the data life cycle [22]. Comparative databases are offered by the European Bioinformatics Institute EMBL‐EBI [23], and databases for specific content, such as BRENDA for enzymes or RCSB PDB [18], UniProtKB for proteins [24], or GenBank for genomic data provide discipline‐specific knowledge [11]. However, a known problem is that content‐specific databases are often not interconnected to ensure processing of complex queries [18]. For example, when studying enzymes, one needs to assemble the protein structure or genetic code from different databases and repositories. Although life sciences are known to be willing to share data, standardization or universal interfaces for data could provide more transparency in life sciences [5, 19]. However, even in the life sciences, there are often proprietary data such as toxicity data, molecules of drug candidates, or pharmacological test data that are not publicly available.

In engineering science, many standards are already set by the technology industry [25]. In engineering, access to open databases and repositories is more limited than in the life sciences [25]. Therefore, to make research data FAIR in engineering, there have been recent initiatives to involve all areas of engineering research, as well as experienced infrastructure providers and initiatives such as NFDI4Ing, to make data management FAIR [26]. Particular challenging were proprietary data and patents if limited accessible and not publicly available, or most engineering data were held in specific data silos to serve particular topics within the discipline, for example, thermodynamics or energy topics [25].

Cross discipline science like bioengineering has to meet a different mindset, tools and solutions for research [27]. New mindsets are also needed in data management, and often data management issues are not conclusively resolved. For example, it has been found that only half of researchers had documented protocols for backing up and storing files [15]. While there are few standards in the life sciences but very many databases and repositories [5, 9], the opposite is true in engineering, where there are many standards but few repositories [5]. The challenge for biotechnology is to find overlapping areas for both sciences and to map them in data management. The majority of researchers in biotechnology works on broad research topics from many overlapping areas [28]. In this context, the generation of numerous and heterogeneous data sets often leads to the use of different heterogeneous file storage solutions and different discipline‐specific practices [19, 29] to store, for example, process control and monitoring or growth and process kinetics data in bioreactors. This necessitates full end‐to‐end data exchange and integration of process data such as scale‐up/down conditions in combination with biological parameters [30]. Data Platforms used in biotechnology often have to make trade‐offs [29]. In this context, general repositories are best suited for cross‐disciplinary research in biotechnology, such as DRYAD Berkley [31] or Dataverse, DaRUS [32], which allow both disciplines equal sustainable data management but excluded data silos.

As an integral part of the “InterZell” priority program, the Dataverse DaRUS has already been available to the 50 biotechnology researchers from the fields of life sciences, process optimization and engineering since the beginning of the research network. The research focus of the priority program is on cell–cell or cell–bioreactor interactions, in which enormous amounts of research data (Big Data) will be generated in experiments or in simulations and modeling. In order to structure these data in a FAIR‐compliant manner, the coordinator provided the prerequisites in the initial phase of the program. Therefore, a concept was created how the life cycle of the data can be structured (Figure 1A), in order to subsequently structurally adapt the data management to the requirements of the program (Figure 1B). It became apparent that the main and thus central role had to be performed and managed by a coordinator as admin (Figure 1B), who is responsible for providing the infrastructure for the data and for offering the required training to the team. To ensure that the infrastructure was in position at the initial stage, even though no data was available at that time, the structure was specified from top to bottom. Subject‐specific criteria such as metadata, etc., were defined by the coordinator together with the experts (Figure 1B). However, it became apparent that to implement the FAIR principle, each researcher in the program had to act proactively and independently. To make the data available FAIR, each research group can independently access the provided infrastructure after training and empowerment. Specific levels of access were established for researchers. However, in order for the data to be published, it requires the approval of the principal investigators. Thereafter, the published datasets are citable, FAIR compliant and provide specific biotechnology discoverable metadata, field relevant community standards. As a result, the cross‐disciplinary structure of the bioengineering research network becomes usable as a community approach with verified sustainable research data.

**FIGURE 1:**
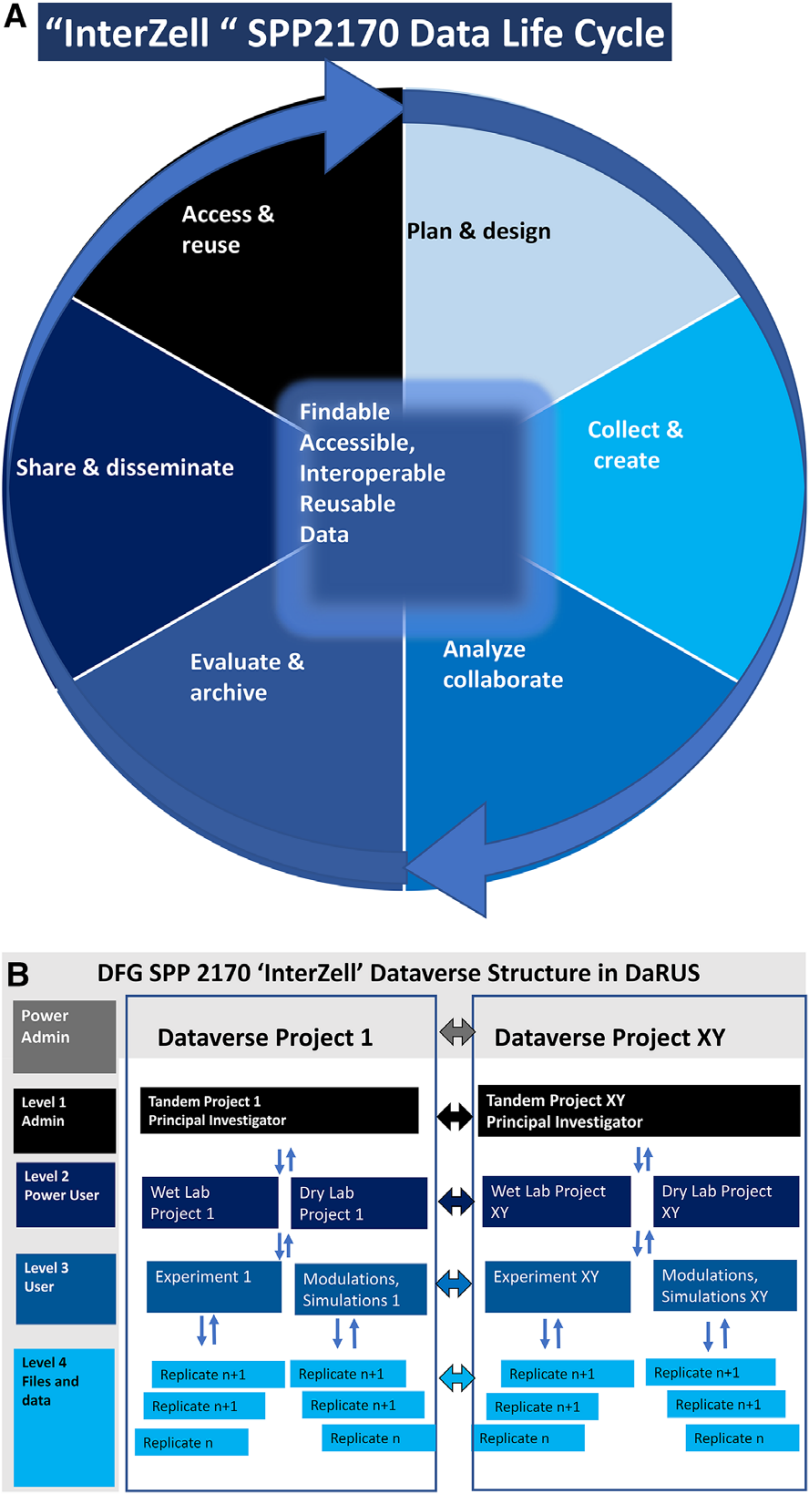
Framework for SPP2170 priority program data management proposed throughout the data lifecycle. Top‐down strategies for RDM structure and bottom‐up strategy for data storage. (A) SPP2170 data lifecycle model: plan and design, collect and create, analyze and collaborate (light blue), evaluate and archive, share and disseminate, access and reuse (dark blue), adapted to Harvard education [3]. (B) Mapping “InterZell” requirements to the Dataverse structure in DaRUS. Different levels and protocols for storage, publication and revisions in the priority program have been established. The structure was implemented in the repository DaRUS for the SPP 2170 “InterZell” with metatags and special member groups defined for dry lab/simulation and wet lab requirements. Top‐down implementation of the structure according to data life cycle by the power admin (coordinator). The researchers (level 2–4) will be responsible for bottom up data storage, and the principal investigator for access and reuse (level 1)

A top‐down strategy could support FAIR data management [2] from the beginning of a project, However, this can only be accelerated by responsible persons, such as coordinators, who establish the top‐down strategy from the beginning by decision‐making on structure, design, resources and funding for a reliable data infrastructure. But the implementation of FAIR data management also requires researchers and their acceptance to deposit data according to the RDMs, however, performed bottom up [34]. Therefore, transparent operational data management requires both a top‐down strategy in advance by responsible parties and a bottom‐up strategy during the life cycle of the data by the researchers involved [29, 35].

Cross‐disciplinary collaborative approaches in biotechnology require universally accessible repositories open to all disciplines worldwide. The DaRUS repository met these conditions and was therefore applied to the “InterZell” program. However, FAIR data management in biotechnology can only be successful as a collaborative approach. RDM, which started with a top‐down strategy, that is, providing infrastructure and training, will fail if it is not accepted by teams as part of a bottom‐up strategy. A collaborative approach requires that all researchers manage their data FAIR throughout the lifecycle.

As a cross‐cutting discipline, data management in bioengineering must meet the different requirements of the life sciences and process engineering, and must therefore be continuously adapted as the two individual disciplines evolve. Therefore, responsible experts, coordinators, and data curators are needed to continuously adapt the structure of data storage to meet the challenges, especially in these heterogeneous frontier areas. However, RDM is only effective in everyday research if FAIR is accepted as a collaborative community approach.

## CONFLICT OF INTEREST

The authors have declared no conflicts of interest.

## Data Availability

The requirements that support the findings of this study are available in the dataverse DaRUS at https://doi.org/10.18419/darus‐829, reference number [10.18419, 829] SPP2170_InterZell_DMPV1_1, on request. Data sharing not applicable to this article as no datasets were generated or analyzed during the current study.
